# Supplementary figures and images for: Microsaccades strongly modulate but do not directly cause the EEG N2pc marker of spatial attention
Source: PLoS Biol. 2025 Sep 25;23(9):e3003418. doi: 10.1371/journal.pbio.3003418 (PMC12500154; doi:10.1371/journal.pbio.3003418)

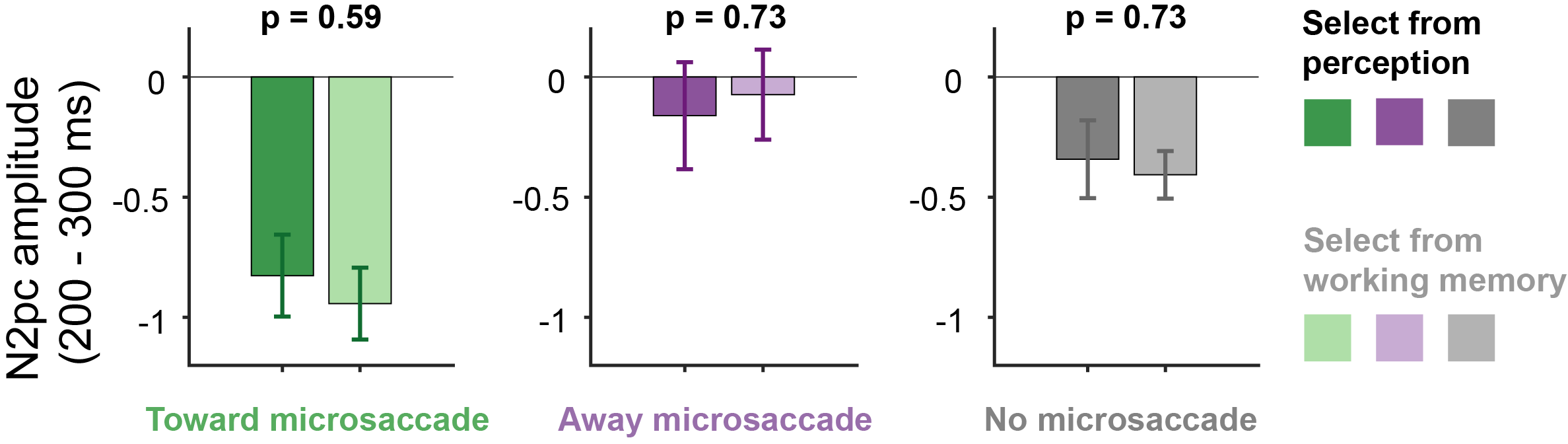

Supplement: S1 Fig — (TIF) [file pbio.3003418.s001.tif]

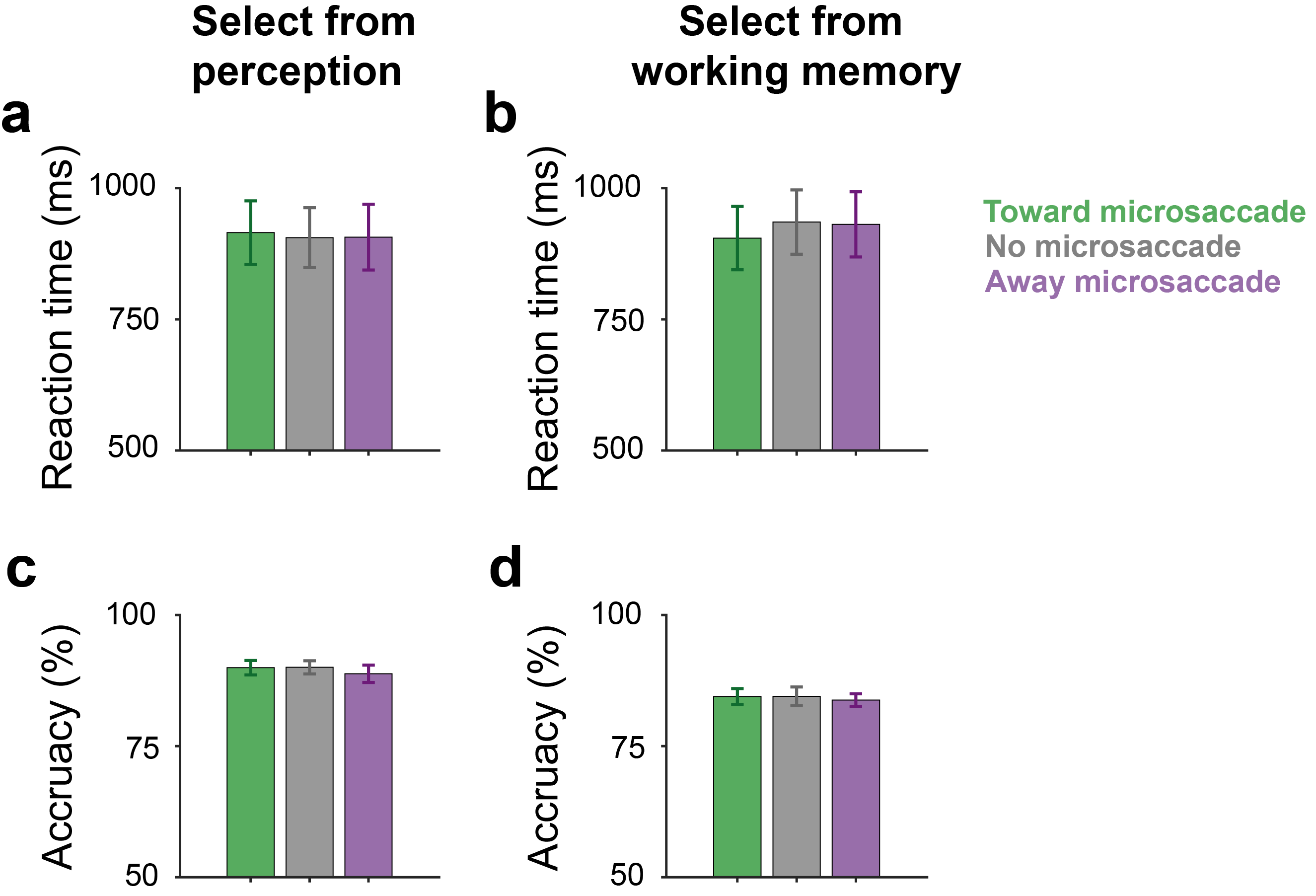

Supplement: S2 Fig — The mean of reaction time (a, b) and the mean of accuracy (c, d). Bar graphs show mean values, with error bars indicating ±1 SEM calculated across participants (n = 23). For reaction time, a one-way ANOVA did not yield an effect of microsaccade trial-class in the perceptual-selection task (F(2, 44) = 0.2, P = 0.82, partial η2 = 0.009), but did show a main effect in the working-memory selection task (F(2, 44) = 6, P = 0.005, partial η2 = 0.21). Post-hoc t test in the latter task showed how reaction times in toward-microsaccade trials were shorter than in both no-microsaccade (t(22) = −3.89, PBonferroni = 0.002, d = −0.81) and away-microsaccade trials (t(22) = −2.82, PBonferroni = 0.03, d = −0.59). No significant difference was found between no-microsaccade and away-microsaccade trials (t(22) = 0.4, PBonferroni = 1, d = −0.08). For accuracy, one-way ANOVAs did not yield a significant effect of microsaccade trial-class, neither in the perceptual-selection task (F(2, 44) = 1.26, P = 0.29, partial η2 = 0.05) nor in the working-memory selection task (F(2, 44) = 0.28, P = 0.76, partial η2 = 0.01). (TIF) [file pbio.3003418.s002.tif]

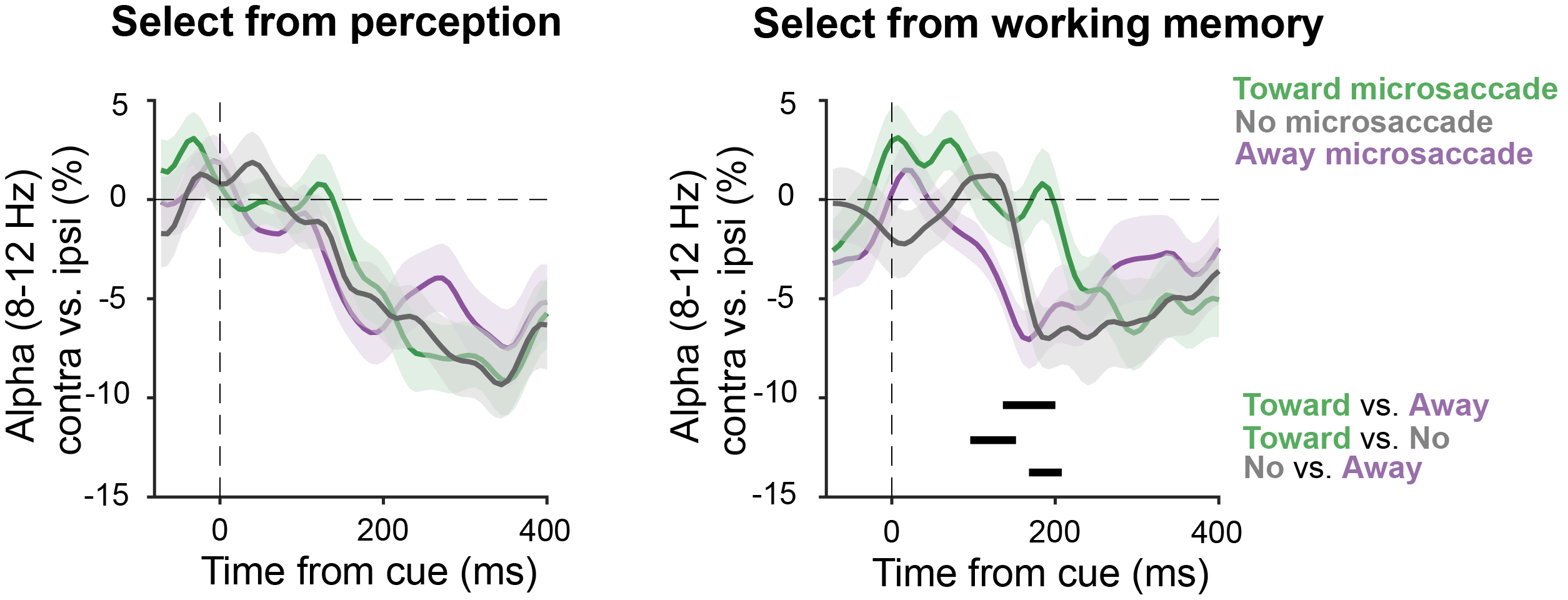

Supplement: S3 Fig — Black horizontal lines indicate significant temporal clusters for the comparisons indicated on the right (two-sided cluster-based permutation test). Time courses show mean values, with shading indicating 95% SEM (calculated across 23 participants). (TIF) [file pbio.3003418.s003.tif]

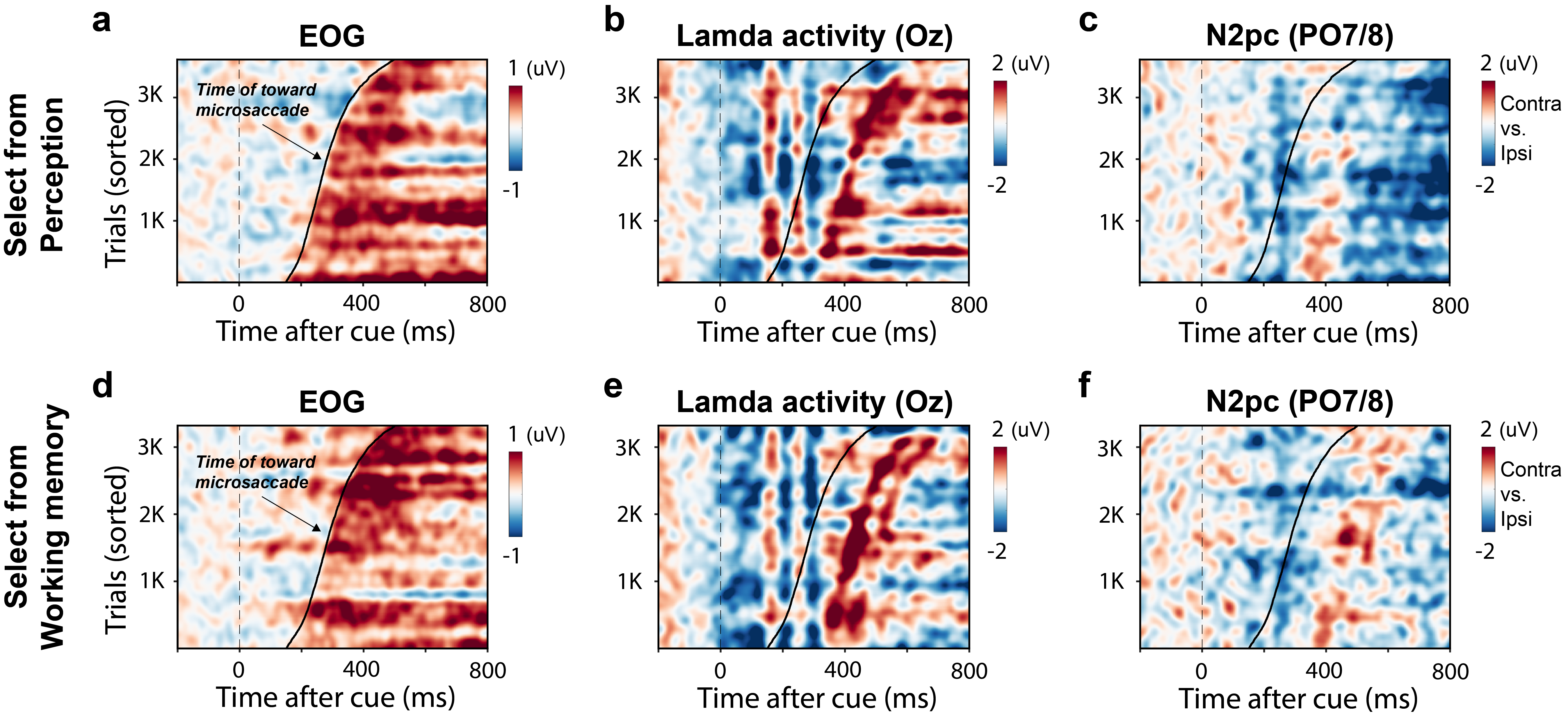

Supplement: S4 Fig — (TIF) [file pbio.3003418.s004.tif]

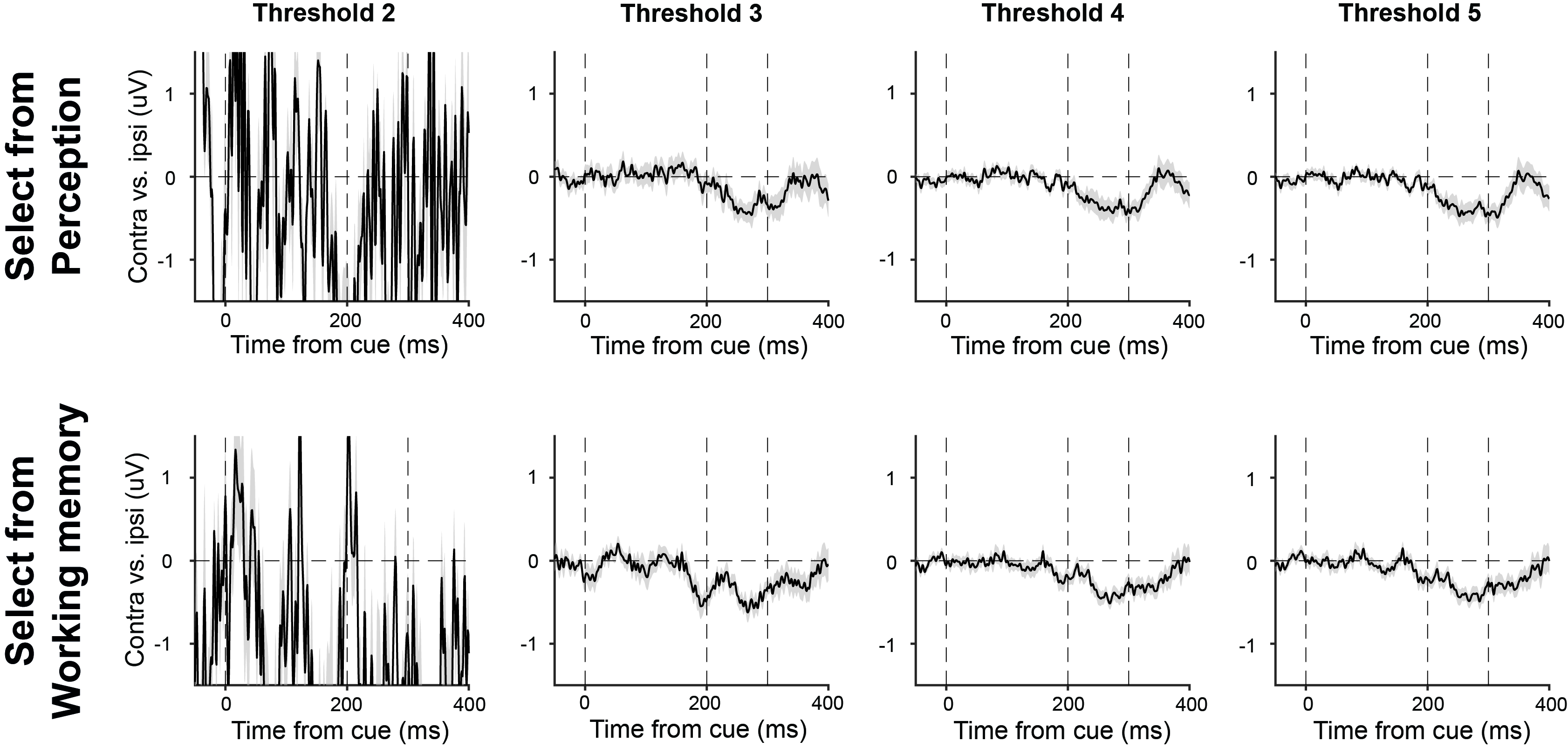

Supplement: S6 Fig — We compared ERP results in the “no-microsaccade” condition using different microsaccade detection thresholds (2, 3, 4, or 5 times the median gaze velocity). Waveforms reflect the contralateral-minus-ipsilateral difference at PO7/8. The top row shows results for the “select from perception” condition; the bottom row shows results for the “select from working memory” condition. A threshold of 2 (times the velocity) results in unstable and noisy ERPs. Thresholds of 3 (times the velocity) and above yield consistent and interpretable N2pc effects. Shading indicates ±1 SEM across participants (n = 23). (TIF) [file pbio.3003418.s006.tif]

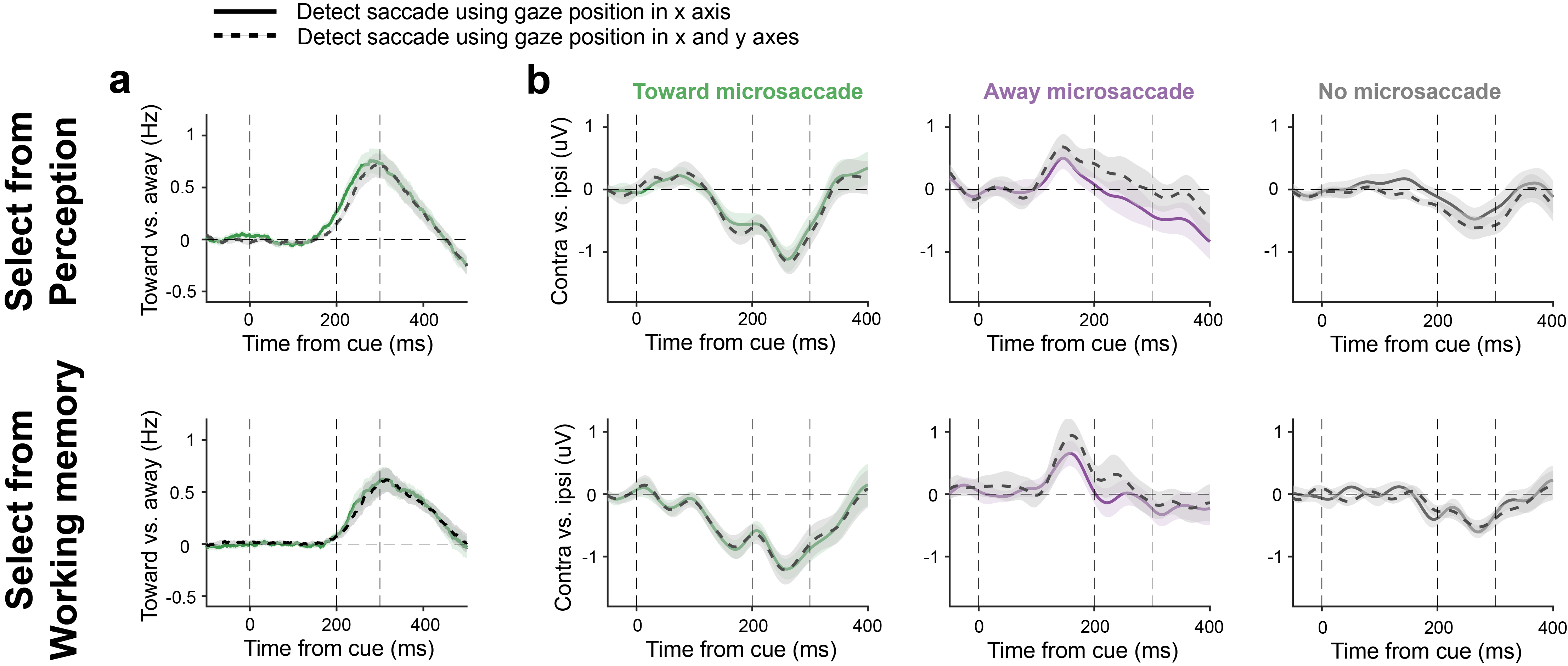

Supplement: S7 Fig — We re-ran our microsaccade-detection approach using a 2D velocity vector, combining horizontal and vertical gaze positions by calculating the 2D Euclidean distance between temporally successive samples. The remaining steps in the detection pipeline (including smoothing, thresholding, onset identification, direction classification, and magnitude filtering) were kept identical to the original 1D method. (a) Time course of spatial bias in microsaccade direction, detected using either 1D (solid line) or 2D (dashed line) velocity-based methods. (b) N2pc waveforms (contra versus ipsilateral ERPs in PO7/8) for trials categorized by microsaccade direction (Toward target, away from target, or no microsaccade), using both detection methods. Shading indicates ±1 SEM calculated across participants (n = 23). Colored lines correspond to microsaccade detection using 1D data; gray dashed lines represent detection using 2D data. (TIF) [file pbio.3003418.s007.tif]
