# Supplementary material for: Microsaccades strongly modulate but do not directly cause the EEG N2pc marker of spatial attention
Source: PLoS Biol. 2025 Sep 25;23(9):e3003418. doi: 10.1371/journal.pbio.3003418 (PMC12500154; doi:10.1371/journal.pbio.3003418)
Supplement: Attachment — Submitted filename: RepliesToReviewers_Liu_Kong_vanEde.docx [file pbio.3003418.s010.docx]

We thank the reviewers for their constructive and thoughtful feedback on our manuscript. We were encouraged by the recognition of the importance and novelty of our work. We were also grateful for the valuable feedback that we have embraced to strengthen our article. Prompted by this valuable input, we have including the outcomes of several additional analyses that corroborate our conclusions and that substantially increase the comprehensiveness of our contribution. Below, we respond in detail to each point raised.

**Reviewer 1 (Leon Y. Deouell)**

The manuscript by Liu, Kong and van Ede examines whether the N2pc, a commonly used lateralized EEG measure of spatial attention, depends on the occurrence of microsaccades, small saccades occurring involuntarily while attempting fixation. The question is appropriate - several studies in the last decade or so have shown that while attem pting to maintain fixations (i.e. when subjects are required to not move their eyes), microsaccades are nevertheless biased in the direction of spatial attention. Moreover, several previous studies have shown that EEG measures (ERPs and spectral components) can be affected by the occurrence of microsaccades.

Here, Liu et al. presented subjects with cued spatial attention tasks, requiring the subjects to maintain fixation while awaiting a probe relating to either the left or right stimulus. A central cue indicated which side is to be probed. An interesting twist was that in one condition the left and right stimuli remained on the screen when the side to be attended was cued, while in another condition, the stimuli were gone by the time the cue appeared, requiring a shift of spatial attention over memoranda. As expected based on the above mentioned studies, microsaccades were strongly biased towards the cued side. The N2pc was robust when subjects made a saccade towards the attended side, and absent when they made a saccade in the other direction, suggesting a strong modulation. The critical point is what happens in trials in which no saccade was made (a bit over a 1/3 of the trials). Here, the authors argue that there is still a small but significant effect, which drives them to the main argument reflected in the manuscript title.

Overall, this study presents an important cautionary note about the importance of careful examination of eye movements in EEG experiments, especially when lateralized stimuli are presented (but not only, see Yuval-Greenberg et al., 2008, 2009; Dimigen et al., 2009). The results are quite striking (but see questions about the interpretation below) and the inclusion of both the perceptual and the memory-based task is elegant.

Thank you for appreciating the importance of our question as well as the use of both a perceptual and a working-memory task in our study. Thank you also for your valuable comments that have helped us to substantially improve our contribution by: (1) verifying the suitability of our chosen analysis parameters, (2) adding the outcomes of additional analyses to clarity the nature of the reported relation between spatial microsaccade biases and the N2pc, and (3) adding relevant clarifications and discussions throughout our article. We elaborate on these points in our point-by-point replies below.

Major comments:

1. The robust effect in this study is that absent a microsaccade in the direction of the attended side, the N2pc is greatly reduced, and thus the argument for the residual effect requires scrutiny. This is especially the case since in one of the conditions (the perceptual one), the N2pc is feeble, and is not significantly different from the away-microsaccade effect. This requires careful examination of the processing stages to determine if there were truly no microsaccades in the so-designated bin:

a. Was only the right eye measured? The Eyelink 1000 allows for a binocular measurement, and especially for microsaccades, the correlation between the eyes is relevant (See Kliegel's algorithm). If only the right eye was actually measured, was it the dominant eye for all subjects?

Thank you for prompting us to specify this more clearly. We only recorded one eye (which was by default the right eye). We now state this explicitly on page 13. In our prior paper, we previously validated this chosen approach by explicitly comparing our approach to the mentioned algorithm from Kliegl et al., using two eyes (afforded by the fact that in that previous dataset we had binocular recordings). Back then we established how our monocular method was at least as sensitive and reliable. For details, see Supplementary Figure 7 in Liu et al., Nature Communications 2022 “Functional but not obligatory link between microsaccades and neural modulation by covert spatial attention”.

We did not assess ocular dominance, but simply resorted to the right eye by default. Across many recent eye-tracking studies from our lab with a focus on microsaccades, this has given us robust microsaccade biases by covert and internal attention that we were also interested in here.

b. What happens if both the horizontal and vertical signals are used when calculating velocity, instead of only the horizontal? Does it make a difference?

Thank you also for this relevant question. Our decision to rely on the horizontal component of gaze was another choice that we previously validated. We fully agree that is worthwhile to also asses the relevance of this choice here. To this end, we now re-ran our microsaccade-detection approach using a 2D velocity vector (combining horizontal and vertical gaze positions by obtaining velocity profiles by taking the 2D Euclidean distances between timepoints). As we show below, using 1D velocity and 2D velocity profiles to detect microsaccades shows nearly equivalent sensitivity for detecting he spatial biases in microsaccades in our task with left and right objects. Moreover, when using 2D velocity to detect microsaccades and sort our trials, our key results concerning the modulation of the N2pc by microsaccade direction and presence are preserved.

Because we believe these are valuable re-assuring findings that corroborate our conclusions, we have now included them in our article:

Page 13 (Method)*:*

*“*In addition, we confirmed that the results remain consistent when using a 2D velocity vector that combines both horizontal and vertical gaze components for microsaccade detection (see **Supplementary Fig. 6**).*”*

Page 25 (Supplementary Material):

**Supplementary Figure 6. Consistent results when detecting microsaccades using 1D (horizontal) versus 2D (horizontal and vertical) gaze data.** We re-ran our microsaccade-detection approach using a 2D velocity vector, combining horizontal and vertical gaze positions by calculating the 2D Euclidean distance between temporally successive samples. The remaining steps in the detection pipeline (including smoothing, thresholding, onset identification, direction classification, and magnitude filtering) were kept identical to the original 1D method. **a**) Time course of spatial bias in microsaccade direction, detected using either 1D (solid line) or 2D (dashed line) velocity-based methods. **b**) N2pc waveforms (contra vs. ipsilateral ERPs in PO7/8) for trials categorized by microsaccade direction (Toward target, Away from target, or No microsaccade), using both detection methods. Shading indicates ±1 SEM calculated across participants (n = 23). Colored lines correspond to microsaccade detection using 1D data; grey dashed lines represent detection using 2D data.

c. The permissive velocity threshold seems like a justified conservative decision. But how do the results change if you change the velocity threshold up or down (a multiverse result would be helpful to determine the effect of such decisions)?

This is indeed another relevant analysis parameter. Indeed, it is important to set a low threshold to arrive at the most trustworthy ‘no-microsaccade’ condition (at the inevitable expense of potentially over-classifying noise as microsaccades). Accordingly, we deliberately set a low threshold (3 x median) in this study, compared to more established defaults of 5 x the median (which is also the default in our function, as well as a widely adopted threshold in the wider literature). Prompted by this valuable comment, we now also conducted an analysis using different velocity thresholds (2, 3, 4, or 5 times the median velocity) to define microsaccades. As the below figure shows, a threshold of 3 appears the lower bound for meaningful EEG analysis. Because this is a very low threshold, we remain confident to label trials without a threshold crossing in the key window of interest ‘no microsaccade’ trials.

Because we believe these are relevant additional results, we have now also added these additional results to our article:

Page 13 (Method):

“To further validate this choice, we conducted additional analyses using a range of thresholds, confirming that a threshold of 3 times the median gaze velocity was a lower bound for retaining sufficient trials to yield interpretable ERPs (see **Supplementary Fig. 5**).”

Page 24 (Supplementary Material):

**Supplementary Figure 5. N2pc waveforms in trials classified as “no-microsaccade” under varying velocity thresholds.** We compared ERP results in the “no-microsaccade” condition using different microsaccade detection thresholds (2, 3, 4, or 5 times the median gaze velocity). Waveforms reflect the contralateral-minus-ipsilateral difference at PO7/8. The top row shows results for the ‘select from perception’ condition; the bottom row shows results for the ‘select from working memory’ condition. A threshold of 2 (times the velocity) results in unstable and noisy ERPs. Thresholds of 3 (times the velocity) and above yield consistent and interpretable N2pc effects. Shading indicates ±1 SEM across participants (n = 23).

At the same time, we agree that this is a vital point to remain critical of and we now more explicitly raise this in our discussion:

Page 10 (Discussion):

*“*Our interpretation that co-occurring microsaccade biases are not necessary for the N2pc to be observed hinges on how trustworthy the “no-microsaccade” condition is. We acknowledge that detecting microsaccades with perfect sensitivity and specificity remains a methodological challenge. Nevertheless, we implemented two critical steps to support the robustness of our interpretation. First, to minimize the risk of missing subtle microsaccades, we deliberately applied a low detection threshold (3 times the median gaze velocity), which we identified as the lower bound for producing stable ERP estimates (see **Supplementary Fig. 5**). This conservative approach increases our confidence that trials labeled as “no microsaccade” are indeed free of eye movements in the critical analysis window. Second, […]”

Finally, please also see our new results in response to the point below, where we show that even though microsaccade direction strongly modulated the N2pc, the N2pc is nonetheless not precisely locked in time to the microsaccade (suggesting a more complex relation than a direct ocular/muscle artefact or a direct visual consequence of retinal displacement).

2. I missed an attempt to explain how or why the microsaccades affect the N2pc measurement. In principle, this can happen in at least 3 ways - 1) the change in retinal image caused by the microsaccade can change visual cortical responses 2) the electric potential caused by the movement of the eye and the activity of ocular muscles could confound\distort the desired signal in several ways (see Keren et al., 2010), and 3) the more benign reason for a correlation between microsaccades occurrence and ERPs is that there is a common factor driving both, namely, attention. The authors argue based on the behavioral and alpha results that attention was similarly shifted in trials with no saccades, basically arguing against cause #3. The memory-based condition mitigates cause #2 (lines 262-264) although it should be noted that the cue remains on the screen and becomes lateralized when a microsaccade occurs. This leaves us with option #2. So, is it a matter of signal mixture that makes the attentional N2pc seem larger when microsaccades co-occur? Perhaps a trial-by-trial analysis like the one in Figure 4 of Dimigen et al., 2009 (J Neurosci 29, 12321-12331) or Figure 4 of Yuval-Greenberg et al., 2008 could help?

Thank you for carefully delineating the three possibilities. We fully agree that the sketched possibilities are three viable options (in addition to the possibility that microsaccades and the N2pc may be completely unrelated, of course). As the first response to your important comment (as well as a related comment of R3), we have now expanded our introduction to explicitly outline these different possibilities:

Page 3 (Introduction):

*“*We considered four ways in which spatial biases in microsaccade directions and the N2pc might be related. First, microsaccades and the N2pc may be unrelated. Second, because microsaccades cause small shifts in retinal input that drive visual cortical activity [35,36], spatial biases in microsaccades may impose spatial modulations of the EEG. Third, the engagement of eye-muscles during microsaccades may directly introduce artifacts [37,38] that may also lateralise according to microsaccade direction. Finally, microsaccade direction and the N2pc may both be modulated by a common underlying factor, without microsaccades *directly* causing the N2pc (as recently postulated for neural signatures relevant to attention [22,33]). Here we provide evidence for a profound modulation of the N2pc by spatially biased microsaccades that is most consistent with the fourth scenario*.”*

We also wish to express our gratitude for prompting us to run additional analyses to better clarify and visualise the reported link between microsaccades and the N2pc. As we show below, these additional analyses and visualisations portray an even more intriguing story on the link between spatial biases in microsaccades and the N2pc, while also ruling out the low-level ‘eye-muscle artifact’ and ‘retinal displacement’ interpretations.

Specifically, we have now calculated the “ERP image” of the N2pc, where we focused on trials with toward microsaccades that we sorted according to the timing of the toward-microsaccade onset. The logic is as follows: if toward microsaccades directly cause the N2pc because of an eye-muscle artifact or retinal image displacement (of the stimuli and/or the fixation marker/cue), then the N2pc should be precisely locked in time to the timing of the toward microsaccade. Critically, this is not what we observed – despite the observation that trials with vs. without toward microsaccades so clearly differ in N2pc amplitude. We have now added the outcomes of this revealing analysis to our article as we show below. In addition, this has prompted us to revise our article at several key instances, as we detail below, after showing these informative results:

Page 8 (Results):

***“*Microsaccades do not directly cause the N2pc**

In a final analysis we investigated the temporal correspondence of microsaccades and the N2pc. We reasoned that if the observed N2pc modulation is driven by a direct consequence of microsaccades – due to changes in retinal input or eye-muscle activity – then the N2pc should precisely align with microsaccade onset across trials. To investigate this, we focused on trials that contained toward microsaccades and sorted them by microsaccade onset latency (using the logic of the ERP-image [44]). To increase the sensitivity and robustness of the analysis, we pooled the data form the perception and working-memory tasks (though the results are consistent when analysed separately for both tasks; **Supplementary Fig. 4**).

We first confirmed the validity of our microsaccade sorting. As shown in **Figure 4a**, the EOG showed a clear modulation that was precisely aligned to microsaccade onset. This shows that our sorting worked and that our eye-tracking measurements (from which we extracted microsaccades) and EEG measurements (that contained the EOG channel) were properly synchronized. Similarly, when looking at the non-lateralised ERP in electrode Oz (**Fig. 4b**), we found a clear Lamda response (as in [35]) approximately 100-150 ms after microsaccades that also scales with microsaccade timing.

Critically, however, no such precise temporal alignment was observed for the N2pc – the blue negativity in **Figure 4c** that remains consistently aligned to the moment of the cue, emerging around 200-300 ms after cue onset. Thus, even though microsaccade presence and direction matter a lot for the N2pc (**Fig. 2**), the N2pc is not directly locked in time to microsaccade onset. This rules out that the observed link between microsaccades and the N2pc is due to retinal displacement or eye-muscle artifacts, as such variables would have been expected to yield a precise locking in time (just like we had seen for the EOG and Lambda activity; **Fig. 4a,b**).

**Figure 4. EOG, Lambda, and N2pc activity in toward-microsaccade trials, sorted by the time of microsaccade onset.** Trials containing toward microsaccades were sorted by the latency of microsaccade onset (black line) and plotted as ERP-images for horizontal EOG (**a**), Oz (**b**), and the lateralised N2pc in PO7/8 (**c**). Color indicates voltage (µV). Data were aggregated across all participants and both tasks. ”

Page 15 (Methods):

*“***ERP-image analysis.** To assess the precise temporal relationship between the N2pc and microsaccade onset, we performed an ERP-image analysis [44], focusing on trials containing toward microsaccades. Trials from all participants were pooled and sorted based on the latency of the toward microsaccade in each trial (relative to cue onset). For visualization, the sorted 2D trial matrix (trials × times) was smoothed using a Gaussian kernel (using MATLAB’s built-in “smoothdata” function), applied first across 10% of neighboring trials in sorted order (y axis in the 2D matrix) and then across a 50-ms slide window in the time domain (x axis in the 2D matrix).*”*

To accommodate this new addition to our article, we have also revisited and revised the framing of our article at key instances, including our abstract and discussion, as we detail below:

Page 2 (Abstract):

“Moreover, despite the strong modulation of the N2pc by microsaccade presence and direction, the N2pc does not align to the precise timing of microsaccades, ruling out that the observed N2pc modulations by microsaccades are a direct artifact of microsaccade-related eye-muscle activity or visual inputs moving over the retina. Thus, while microsaccades strongly modulate N2pc amplitude, microsaccades themselves are not a prerequisite for, nor a direct cause of, the N2pc.”

Page 10 (Discussion):

*“*Despite the strong effect of microsaccade presence and direction on the N2pc, our ERP-image analysis (where we sorted the N2pc according to the timing of toward-microsaccades), showed that the N2pc did not align to the precise timing of microsaccade onset. This temporal dissociation suggests that microsaccades do not *directly* cause and/or contaminate the N2pc through low-level mechanisms like retinal displacement or eye-muscle activity, which would both be expected to be precisely locking in time to microsaccade onset. Instead, our findings support an interpretation whereby both the N2pc and microsaccades are driven by a common underlying process (e.g., a component of attention). In this interpretation, this common underlying process yields both the N2pc (whose timing aligns to the cue), and biases the direction of ongoing microsaccades (whose precise timings remain subject to additional factors like build-up of activity in the oculomotor system and refractory periods between eye-movements [45,46]). This fits with the observation that attentional processes do not directly trigger new microsaccades, but only bias the direction of microsaccades that happen to be made around the time of the attentional shift [47]. In contrast, these same attentional processes may still directly trigger the N2pc. Such a scenario, though speculative, would yield a correlation between microsaccade directions (in the overall N2pc time window) and the N2pc, without a strict temporal correspondence at the level of single trials, as we observed here.”

Finally, please note how this revealing additional analyses has prompted us to revise our title to: “Microsaccades strongly modulate but do not directly cause the N2pc marker of spatial attention”.

We are thus truly grateful for your valuable suggestion that we believe has made our findings more complete – and even more intriguing.

3.      Figure 2 reveals an early effect (~150-200ms) where "Toward" and "Away" saccades create opposite asymmetries - that is, stronger negativity contralateral to the direction of saccades, rather than the direction of cued attention. There is a trend in the perception condition and significant effects in the memory condition (in the memory condition this trend is also seen in the 200-300 window by the pinkish blob in the occipital half-scalp in the Away condition). This effect  may also be clarified by the trial by trial analysis suggested above.

Thank you also for this suggestion. We also found this early effect intriguing and have now analyzed it more carefully using the ERP-image approach mentioned above. As shown above, while the toward microsaccade trials indeed appear to have a double N2pc peak with an early and a late component, neither components appear precisely locked in time to the onset-time of the microsaccade. Our follow-up analysis thus indeed helps to somewhat clarify this observation, by ruling out a direct artifact (i.e., and eye-muscle or retinal displacement) associated with microsaccade implementation.

In addition, we now also briefly return to this specific aspect of our results in our discussion:

Page 11 (Discussion):

*“*Potentially related to this point, we also noted an earlier lateralized EEG signal (~150–200 ms after cue onset) that preceded the classic N2pc time window and that varied with the direction of microsaccades, independent of side of the cued object (**Fig. 2b,f**). This may possibly be related to the N170 response (for relevant prior work relating microsaccades to the N170, see [55,56]), though further work is needed to unpack this possibility.*”*

Minor comments
4. A discussion of the difference between the perceptual and memory conditions is warranted. What could be the reason for the finding of N2pc with no microsaccades in the memory condition and not so much in the perceptual condition (in the same subjects)?

Thank you. We primarily included the two conditions to help generalise the results and to get a first handle on potential retinal contributions of attended stimuli themselves moving close to the fovea with toward microsaccades (in perception but not working memory). That said, we agree it is also interesting to compare the two settings directly. To this end, we now also directly compared the N2pc between the perception and working-memory cases, separately for each microsaccade trial-type. Despite the apparent differences (e.g., with a no-microsaccade cluster surviving in the working-memory task but not in the perceptual task), we did not observe any statistically significant differences in the N2pc when directly comparing the perception and working-memory task variants in any of our trial classes, as we show below. Because we agree this is another relevant piece of data, we also included the outcomes of these additional analyses to our article:

Page 7 (Results):

*“*For completeness, we also directly compared the microsaccade-related N2pc modulations between the perceptual and working-memory task (**Supplementary Fig. 1**). We observed highly comparable N2pc amplitudes in both tasks for each microsaccade class (all Ps > 0.5), suggesting a similar modulation in both tasks. Thus, the observed N2pc modulations by microsaccades hold regardless of whether microsaccades bring attended visual targets physically closer to the fovea (as in perception) or not (as in working memory).”

Page 20 (Supplementary Material):

**Supplementary Figure 1. Comparison between the N2pc from perceptual and working memory tasks that are separately shown in the main Figure 2.**

5. It is up to the editors to decide, but I would prefer to have the supplementary figures in the main text. They are important enough in my opinion.

Thank you for appreciating the value of our supplementary figures. We initially decided to put these additional findings in our supplementary material to not detract from the focus on the N2pc, and to adhere to the short-report formatting standards. While remaining mindful of these formatting standards, we now brought the alpha-lateralisation figure into the main article (that we believe most clearly shows how spatial attention was shifted even in no-microsaccade trials).

(In addition, please now how we also included the new ERP-image figure presented above as a new main figure in our article.)

**Reviewer 2**

This is a nice and clearly written paper with nice graphics. It also touches on an important topic.

Thank you for calling out the importance of our contribution, and for appreciating our write-up and visualisations.

I have some comments below, primarily related to placement in the proper context of the literature, and also with suggestions to avoid getting bogged down by some unnecessary debates:

Thank you also for these valuable suggestions that have helped us to better situate our findings in the relevant literature and to avoid unnecessary debates, as we detail in a point-by-point manner below.

-Lines 26, 27: what if there are unrealized microsaccade programs? i.e. motor preparation (this is elaborated on a bit in a later comment).

Thank you. As we detail in response to the related comment below, we now explicitly discuss this interesting possibility in our article.

-Line 43: Microsaccades are not involuntary! See Willeke et al., Nat. Comm., 2019, which also explains the long history of this misconception.

Thank you for reminding us of this relevant point. We have removed all mentions of microsaccades as being involuntary.

-Lines 48, 49: It should be added that this is a relevant point and not just theoretical, especially given work like: Hafed, Neuron, 2013; Hafed et al., Front. Sys. Neurosci, 2015; Tian et al., Front. Sys. Neurosci., 2016; Chen et al., Curr Biol, 2015; Zhang et al., BioRxiv, 2024. I.e. this is something that already has some support, and therefore warrants further investigation in this study and future ones

Thank you also for reminding us of this relevant prior relevant literature that we agree helps to motivate our question. We now bring this impressive body of research forward as another key element motivating our question.

-Line 53, for the references 22, 27, 28, there's also Chen et al., Curr Biol 2015

Thank you, we now also cite this relevant paper.

-Lines 56-58: is this really the case? If the memory representation is embedded in visual maps, then a retinal refresh could still differentially modulated an embedded memory representation. Indeed, eye movements strongly modulate sensory maps even in the absence of sensory drives. So, if there's something else embedded in these maps, then they can be modulated

Thank you, this is a relevant point. We agree that a representation of the visual memory content may still move over retinotopic maps with every eye-movement. We have now revised all relevant phrasing related to this point by referring to “physical movement over the retina”. For example:

Page 3 (introduction):

“In contrast, when shifting attention to visual contents held in visual working memory, the potential contribution of physical displacement of the attended object over the retina is sidestepped because the attended object is in memory.”

-The legend of Fig. 1d (and the main text) are too terse and sparse about what is being shown. Since this is a multi-disciplinary journal, what is written and shown in Fig. 1d is currently simply incomprehensible for people outside the field. For example, what is the purple and what is the green? And how is this indication of "posterior" specificity? And what is the difference between left vs. right and contra vs. ipsi? And, why is left so purple? And what is the color bar in the first place? Too many details are completely missing. Panels b and c are comprehensible, but d is just too abstract. Similarly for k

Thank you for these valuable reflections and our apologies for not stating these relevant details clearly enough. We agree our figure caption for panels d and k was too compressed. We now expanded the caption to cover the above points.

Page 5 (Results):

*“***d, k**) The topographies in the top show the voltage difference between trials where the left object was attended versus trials where the right object was attended, across all electrodes, in the N2pc time window. The bottom topographies show the difference between contralateral and ipsilateral activity relative to the attended side, collapsed into the right electrode of all symmetric electrode pairs. Green shows stronger contralateral negativity signaling the N2pc.*”*

-Line 200: I'm a bit confused here. You're using a brain signal as the "objective" measure ("marker") that attention was deployed. But, the current paper is already showing clearly that this is dangerous, since the N2PC component is clearly microsaccade-related and practically non-existent with attention shifts. Thus, the N2PC "marker" was already proven to not be a marker, and this could also be the case for other markers. So, isn't this circular logic????

Thank you for this sharp comment. Our argument that spatial attention was in fact employed, even when we did not observe a clear N2pc, is based on a combination of behavioural performance data as well as this second marker that ample prior research has linked to attention shifts. Our reasoning is as follows: if the cue was simply not used in these trials, we should not see evidence for any spatial modulation in neural activity. However, since we find clear spatial alpha lateralisation even in these trials (as well as highly accurate task performance) our data suggest the cue was on fact used in these trials, even if this did not show in toward microsaccades nor in the N2pc.

-For me, Fig. 2 is convincing that it's essentially all explained by the towards microsaccades. The small cluster that emerges in the memory condition is qualitatively drastically very different from the classic N2PC, e.g. later, and oscillatory, etc etc etc. I understand that the authors are treading a bit carefully, but I see from this paper that basically all of the N2PC effect is explained by microsaccades, and not just "most". The stuff happening later with very different dynamics (e.g. oscillatory) etc could be explained by other things completely, including (but not exclusively) unrealized motor plans

Thank you for this appreciation of our main finding that microsaccades matter a lot for the N2pc to be observed. At the same time, note how we did still observe a significant N2pc in the no-microsaccade trials. Moreover, in a novel analyses prompted by R1 and R3, we now show how, even though microsaccades so strongly modulate the N2pc, the N2pc is not directly triggered by microsaccades, as they are not aligned one-to-one in time (for details, see our elaborate response to R1-comment 2). This provides further support for a more nuanced interpretation, whereby the presence of toward microsaccades strongly modulate the N2pc, even if microsaccades do not *directly* *cause* the N2pc. In addition, note how these new data also rule out direct muscle-artifacts or retinal displacement as explanations for the reported link.

-Line 226: really? See the above comment. Also, there's always the idea that there was a microsaccade plan that was still not realized. I think that this is a distinct possibility. I know that this sounds like I'm somehow rooting for microsaccades. However, this is exactly the kind of argumentation that people rooting for attention use in order to discount an important role for microsaccades in attention. So, if they can do it there, then it can also be done here. Of course, I don't mean to just do it for the sake of doing it (see later comment about "threat"). What I mean is to consider all possibilities with an open mind

Thank you. We very much agree that an open mind is exactly what is needed when addressing these important questions. The possibility of unrealised microsaccades is an important point, that we now explicitly discuss:

Page 11 (Discussion):

*“*This indirect link with the N2pc, as we observed at the level of oculomotor output (microsaccades), raises an interesting question regarding the link between the N2pc and neuronal activity upstream in the brain’s oculomotor system that may remain below threshold for microsaccade execution (e.g., activity associated with unfulfilled microsaccades or microsaccade plans). In future work, it will be important to supplement our approach with direct recordings in the superior colliculus (cf. [33,33]) to assess the link between the N2pc and neuronal activity in circuits that govern microsaccade generation [49].*”*

-Line 228: this is not the complete story with these citations. For example, see the Hafed lab results mentioned above. Also see the latest Hafed lab work on this topic: Zhang et al., BioRxiv, 2024. In that work, they seem to try to make sense of all of this fuss in their discussion.

Thank you. We now incorporated citations to these important set of studies, included the recent BioRxiv paper that we read with great interest.

-Line 243: again, this "marker" is problematic. What if someone later finds that it's also "severely weakened" by oculomotor activity? I would much rather see mechanistic explanations of how things happen in a brain as opposed to sticking to labels like attention, but that's just me. I mean, no one would ever debate that eye movements strongly modulate brain activity. So, how come we suddenly have to shy away from this when the eye movements are small???? The authors might have an opportunity to make this point clearer here in this manuscript

Thank you. As we already alluded to in response to a related comment above, the primary purpose of these additional results was to show how the complete lack of an N2pc in the no-microsaccade condition was not due to a complete lack of using the cue in these trials. Indeed, if the cue was not being used in these trials, we should have seen deteriorated task-performance, and we should not have seen *any* spatial modulation in neural activity. The spatial modulation of alpha activity (together with high task performance) thus helps us to rule out a lack of cue use (or “lack of attention”) in these trials. This is the primary point served by these additional results.

-Line 276: if someone views a brain phenomenon as a "threat", then they simply should not be brain scientists at all. Why diminish the good work in this paper by getting into such pettiness? I would much rather make this text as a marveling on the revelations that are allowed by the experiments, and not getting too apologetic because some people feel "threatened". Really, I mean, is science about just protecting one's own work (and job) or about learning something new?

Thank youfor this great reflection. We agree and have removed the framing as a “threat”. This now reads:

Page 11 (Discussion):

“Finally, while microsaccades may be considered a nuisance variable in neuroscience studies where instructed gaze-fixation yields fixed retinal positions of external visual inputs, the spatial microsaccade bias is also an interesting signal that brings opportunities for studying attention and working memory [26,57,58].”

-One small minor thing that can be mentioned: there is now increasing evidence that ocular position drifts can also reflect stimulus locations and properties. So, it might be nice to add a sentence or two somewhere indicating that a future direction would be to consider the even smaller ocular position drifts. Who knows, maybe they will also account for the remaining weak n2pc in the absence of microsaccades.

Thank you also for this relevant point. We now mention this as another important avenue for future studies:

Page 11 (Discussion):

*“*…Future studies could further extend these approaches to other neural markers of attentional selection, and to other microscale oculomotor behaviors, like ocular tremor and drift.*”*

**Reviewer 3 (Shlomit Yuval-Greenberg)**

This is an valuable study examining the relationship between microsaccades and the N2pc marker of spatial attention. The findings highlight the temporal co-occurrence of microsaccades and N2pc and demonstrate that microsaccades significantly modulate the EEG response. Crucially, the study also claims that microsacacdes are not a prerequiste for the N2pc, suggesting that this component is not an artifact of eye movements.

I find this topic exceptionally relevant and timely. EEG is becoming increasingly popular as a first-choice brain imaging method due to its accessibility and relatively low cost. However, EEG is also highly susceptible to artifacts, including those from eye movements. Demonstrating both the limitations and advantages of the method is a valuable contribution of this study. Therefore, I support its publication in PLOS Biology, but I have a few concerns and believe the paper could be improved.

Thank you for considering our study and findings valuable and for supporting publication in PLOS Biology. In addition, we are grateful for your excellent suggestions that we have embraced to improve our article. Specifically, we have (1) expanded on the possible ways in which microsaccades and the N2pc can be linked, (2) have included revealing outcomes of additional analyses that help to better understand this link; and (3) have conducted follow-up analyses to verify the suitability of our approach. We elaborate on these improvements in our point-by-point replies below.

Please note how several of the raised points overlapped with key comments and suggestions of R1. Accordingly, several of the replies below show considerable overlap with the replies to R1. Where appropriate, we have chosen to also explicitly refer back to our detailed responses to R1.

Major Concerns:

1. The paper could provide a more in-depth discussion of the possible mechanisms linking microsaccades and N2pc, supported by additional analyses. Several potential explanations exist, including: Corneo-retinal dipole artifacts, the spike potential artifact (see Yuval-Greenberg et al., 2009), visual cortical activity (Lambda wave) (see Olaf Dimigen's studies), non-visual activity (such as attentional shifts, oculomotor-related processes, and decision-making processes). A more thorough review of these possibilities in the introduction, along with a discussion of how the current findings fit within this framework, would strengthen the paper. Additionally, ruling out some of these explanations could be supported by further analyses, particularly examining the topographical distribution of the effect. It would be valuable to see whether the ERP topography differs in the presence vs. absence of microsaccades and whether these differences align with known microsaccade-related artifacts.

Thank you for this excellent suggestion. We have now expanded the introduction to explicitly outline the different possible mechanisms linking spatial biases in microsaccades and the N2pc. This was also prompted by a similar delineation of possible mechanisms put forward by R1 (R1-comment 2):

Page 3 (Introduction):

*“*We considered four ways in which spatial biases in microsaccade directions and the N2pc might be related. First, microsaccades and the N2pc may be unrelated. Second, because microsaccades cause small shifts in retinal input that drive visual cortical activity [35,36], spatial biases in microsaccades may impose spatial modulations of the EEG. Third, the engagement of eye-muscles during microsaccades may directly introduce artifacts [37,38] that may also lateralise according to microsaccade direction. Finally, microsaccade direction and the N2pc may both be modulated by a common underlying factor, without microsaccades *directly* causing the N2pc (as recently postulated for neural signatures relevant to attention [22,33]). Here we provide evidence for a profound modulation of the N2pc by spatially biased microsaccades that is most consistent with the fourth scenario*.”*

Please also note how in response to R1-comment 2, we have now also performed an informative additional “ERP-image” analysis where we sorted our data by the onset time of toward microsaccades. This revealed that, even though the N2pc is most clear in trials with toward microsaccades in the overall N2pc time window, the N2pc is not actually directly caused by microsaccades as evidences by a lack of precise temporal correspondence between microsaccades and the N2pc across trials. This also argues against a direct eye-movement artifact or a direct consequence of retinal displacement of the central fixation cross as we now discuss at the relevant instances in our article. We extensively discuss this new, revealing findings in our response to R1-comment 2, where we also delineate how we have revised our article accordingly.

Finally, please note how we already included the topographical distributions of the N2pc in Figures 1 and 2.

2. The authors claim that the N2pc observed in the working memory condition cannot be attributed to a visual response (Lambda wave) because there is no retinal displacement of the attended object. However, this assumption is problematic. Even without a stimulus on the screen, microsaccades still cause peripheral retinal slips. Given that the experiment was not conducted in total darkness, light from the monitor, its frame, and other elements in the experimental room could produce substantial retinal shifts, leading to visual responses.
This possibility could be ruled out by conducting topographical analyses of the effect using different EEG referencing methods (see Dimigen's studies), or by including a control condition where microsaccades occur without attention deployment (perhaps data recorded during ISIs could be used?).

Thank you. We fully agree that while our working-memory case removes the influence of retinal movement of the attended visual target object, it does not also remove visual movements over the retina from the fixation marker or the screen borders. Thus, while the perceptual and working-memory cases differed in the amount of retinal change related to the attended target, the difference in retinal displacement of any visual input was not all-of-none, but rather a graded difference. Despite this graded difference, we still found near perfect generalisation of our key findings. This suggests that even if we cannot rule out contributions from retinal displacements, retinal displacement alone is unlikely to fully explain the observed effects as that would have predicted a larger modulations in the perception cases where additionally there were visible objects that would move across the retina during eye movements. Still, we agree this is a relevant point, and we have now revised our framing of this, as follows:

Page 10 (Discussion):

*“*While this contrast does not eliminate all visual motion across the retina, since other visual elements such as the fixation marker and screen borders remain, the difference in retinal displacement between these conditions would still be expected to be graded. Despite this, we found near-perfect generalization of our main findings across tasks. This suggests that retinal displacement alone is unlikely to fully account for the observed N2pc modulations, as this would have predicted stronger effects in the perceptual task, where more visual elements are displaced during eye movements. Our dissociation of precise microsaccade and N2pc timing provides further evidence against the contribution of retinal displacement to our findings.*”*

In addition, when alluding to this difference between the working-memory and perception tasks, we now explicitly mention that this difference relates to ‘displacement of the attended object’ rather than displacement of ‘any visual input’. E.g.,

Page 3 (Introduction):

“In contrast, when shifting attention to visual contents held in visual working memory, the potential contribution of physical displacement of the attended object over the retina is sidestepped because the attended object is in memory.”

Finally, to look at this more carefully, and also prompted by R1, we now also calculated the ERP image (with trials sorted by microsaccade onset) as we mentioned in response to the preceding comment. The outcomes of this analysis (that we now added to our article) corroborate the interpretation that the N2pc modulation is not strictly locked to the precise timing of toward microsaccades. This provides more direct evidence against a direct consequence of muscle-artifacts or retinal displacement because those consequences would be expected to be much more precisely locked in time to the onset of microsaccades.

3. Finally, I believe that the conclusion that the N2pc is not caused by microsaccades should be stated more cautiously. The N2pc was abolished when trials with microsaccades in the 150-400 ms window were excluded. The absence of an N2pc cannot be easily attributed to a low number of trials, as no-microsaccade trials comprised nearly 40% of the dataset—similar to the microsaccade conditions. When a shorter 200-300 ms window was examined, the N2pc was only marginally significant, making it possible that residual activity or small undetected microsaccades contributed to the effect. Finally, if Lambda waves were involved, their initial effect would be expected around 100 ms after an eye movement, which could influence the interpretation. A more cautious interpretation acknowledging these uncertainties would make the conclusions more robust.

Thank you for these insightful reflections. We appreciate this caution and have revised our Discussion accordingly as we state below:

Page 10 (Discussion):

*“*Our interpretation that co-occurring microsaccade biases are not necessary for the N2pc to be observed hinges on how trustworthy the “no-microsaccade” condition is. We acknowledge that detecting microsaccades with perfect sensitivity and specificity remains a methodological challenge. Nevertheless, we implemented two critical steps to support the robustness of our interpretation. First, to minimize the risk of missing subtle microsaccades, we deliberately applied a low detection threshold (3 times the median gaze velocity), which we identified as the lower bound for producing stable ERP estimates (see **Supplementary Fig. 5**). This conservative approach increases our confidence that trials labeled as “no microsaccade” are indeed free of eye movements in the critical analysis window. Second, we assessed the precise alignment of the N2pc to the precise timing of toward microsaccades. This revealed that, although microsaccade direction (in the overall N2pc time window) robustly modulated the N2pc, the N2pc was not precisely time-locked to the timing of microsaccade onset in single trials. This temporal dissociation suggests a more complex relationship between microsaccades and the N2pc than would be expected if the N2pc were merely an oculomotor artifact, and thereby provides further support that the N2pc is not directly driven by microsaccades.*”*

In addition, please note how, based on the new analyses discussed above, we now provide further evidence that microsaccades do not *directly cause* the N2pc, as a more direct cause would predict a more precise locking in time of toward microsaccades and the N2pc.

Moreover, while 5 x the median is a common threshold adopted in the literature, we deliberately used 3 x median to increase trust in our critical ‘no-microsaccade’ condition. Prompted by a related comment from R1, we now also conducted an analysis using different velocity thresholds (2, 3, 4, or 5 times the median velocity) to define microsaccades. This verified that our chosen threshold of 3 x median was as low as we could reasonably go, as we detail further in our response to R1-comment1c (Page 3 in this rebuttal).

Finally, regarding the possible interplay with Lambda activity, please note how our newly reported data where we sorted by microsaccade timing (also shown below for convenience) indeed revealed a clear non-lateralised lambda wave in Oz that scaled with the timing of microsaccades. This was distinct from the timing of the lateralised N2pc that was the focus of our study (the blue modulation around 200-300 ms after cue onset in Fig. 4c below). As such, these additional findings (that we now included as a main figure) not only help clarify how microsaccades do not directly cause the N2pc (even if their presence and direction still strongly modulate the N2pc), but also how the N2pc is not a direct consequence of Lamba activity.

**Figure 4. EOG, Lambda, and N2pc activity in toward-microsaccade trials, sorted by the time of microsaccade onset.** Trials containing toward microsaccades were sorted by the latency of microsaccade onset (black line) and plotted as ERP-images for horizontal EOG (**a**), Oz (**b**), and the lateralised N2pc in PO7/8 (**c**). Color indicates voltage (µV). Data were aggregated across all participants and both tasks.

Minor comments:

1. Citations - Olaf Dimigen is a pioneer in this field. He was the first to suggest that ERP components could be influenced by the Lambda wave. His 2009 Journal of Neuroscience paper should be cited and credited in the introduction.

Thank you. We fully agree. We cited three of Olaf Dimigen’s papers in this areas of research, but we now realise that we unintentionally failed to also cite the pioneering 2009 study. We further agree that we should bring this forward in the introduction, as we now do:

Page 3 (Introduction):

“ We considered four ways in which spatial biases in microsaccade directions and the N2pc might be related. First, microsaccades and the N2pc may be unrelated. Second, because microsaccades cause small shifts in retinal input that drive visual cortical activity [35,36], spatial biases in microsaccades may impose spatial modulations of the EEG.”

2. A figure displaying the number of trials per time window for different microsaccade conditions would help clarify the feasibility of comparisons. Alternatively, a random sampling approach could be used to equalize trial numbers across conditions, though this may not be necessary given the already similar trial distributions.

Thank you for this suggestion. It is indeed fortunate that our conditions showed largely comparable trial numbers across the three conditions (albeit with more toward than away microsaccades, which is the effect that sparked our inquiry).

In addition, please note that if our reported N2pc modulations were driven by differences in trial numbers, then we should have expected to see the strongest N2pc in the no-microsaccade trials that had the highest trial numbers. Yet, compared to the no-microsaccades trials, we find a much clearer N2pc in the toward trials, and a lack of an N2pc in the away trials (despite both conditions having less trials than the no-microsaccade condition). Accordingly, the observed differences cannot be attributed to differences in trial numbers. We now bring this relevant point forward at key instances of our article. For example:

Page 6 (Results):

“Note how these differences in N2pc across the three microsaccade classes are unlikely accounted for by mere differences in the number of available trials. For example, while the N2pc more than halved in size when moving from toward- to no-microsaccade trials, we had in fact more (not less) trials in the latter condition.”

In addition, please now how we state the distribution of trials among the three microsaccade conditions on page 6 of our article.

3. The citation of our paper from 2009 (Yuval-Greenberg et al., 2009, Neuron) in reference 40 is inaccurate. That study did not establish a direct link between microsaccades and neural activity but instead demonstrated that oculomotor muscle contractions during microsaccades can appear as EEG signals and be mistakenly interpreted as brain activity.

Thank you for catching this. This is naturally what we meant, but you are right that in your study the microsaccades led to something that people had interpreted as neural activity, but was not. We now cite this more appropriately:

Page 3 (Introduction):

“*…*Third, the engagement of eye-muscles during microsaccades may directly introduce artifacts [37,38] that may also lateralise according to microsaccade direction.”
