# Supplementary material for: Microsaccades strongly modulate but do not directly cause the EEG N2pc marker of spatial attention
Source: PLoS Biol. 2025 Sep 25;23(9):e3003418. doi: 10.1371/journal.pbio.3003418 (PMC12500154; doi:10.1371/journal.pbio.3003418)
Supplement: Attachment — Submitted filename: Liu_Kong_vanEde_RepliesToReviewers.docx [file pbio.3003418.s011.docx]

We are pleased that our three reviewers were each happy with our prior revisions, and we of course also value the few additional suggestions from reviewers 1 and 2. We now also incorporated this additional feedback to further improve the quality and comprehensiveness of our article.

**Reviewer 1**

The revised version of the manuscript went a long way in answering my major concerns. The addition of the sorted trials analysis ("ERP image") illuminates the fact that the lateralized activity (the N2pc) cannot be a simple artifact of the microsaccade, as the N2pc in many cases precedes the saccade onset. If I were the authors, I would emphasize this point. That is, not only that the N2pc does not show up concomitantly or after the saccade, but in some cases it may occur even before. This, together with the added details about microsaccades detection, strengthens the claims of the paper.

Thank you for appreciating our prior revisions, including the added value of our ERP-image results and our clarifications of our microsaccade-detection approach. We remain truly grateful for your valuable inputs that have helped us to strengthen our article. We are also grateful for your additional comments that have helped us to further improve our article, as we delineate below.

We also agree that it is worth placing additional emphasis on how the N2pc can precede microsaccades. We now explicitly state this: “Indeed, for trials with late toward microsaccades, the N2pc often even preceded microsaccade onset.”

That said, the picture will be incomplete without the same ERP image of the "saccades away" trials and "no saccades" trials. In the towards image (right panel of figure 4), there is a clear lateralization (blue) time-locked to the saccade onset with a delay of about 200 ms (see the right side of the right panel in figure 4) - as if each towards saccade elicits an N2pc of its own! What happens in the away saccades? I am not asking just because of curiosity, but because there is still a conundrum remaining - the argument is that a) the N2pc is a genuine signature of attention (and not of eye movements), and b) that attention was equally and adequately shifted in the direction of the cue in the trials with away saccades. These two together predict an N2pc in the away saccade trials, but no evidence of this is found. In the final part of the discussion the authors address this by suggesting some form of 'masking' by summation of potentials on the scalp, but without a more detailed explanation of such masking, it remains mere hand waving. The authors could for example look at (micro)saccade-related-potentials (SRPs) from before the cue, and see if the lateralization of towards vs away saccades (relative to the ensuing cue, but before attention was shifted), can explain why the N2pc seems enhanced with towards saccades and diminished with away saccade. Also, showing the EOG image for the no saccades and away saccades will be illustrative in showing that the saccade detection algorithm is consistent with what the EOG shows. So basically, together with my previous comment, what I suggest is figure 4 to be expanded to show all 3 trial bins (separately).

Thank you also for this valuable suggestion. We have now expanded this figure as proposed and have added this to our article as we show below. Because the away-microsaccade condition has considerably less trials and because the no-microsaccade condition had no microsaccade onset latencies to sort the trials by, we decided to include these additional analyses in our supplementary material and to point to them explicitly when introducing these results (“For a comprehensive overview across toward-, away-, and no-microsaccade conditions, see also Supplementary Figure 5.”). This allows us to be transparent and report our data comprehensively, without detracting the reader from our main take-home-message that is most evident from the toward-microsaccade trials where we also had the clearest N2pc.

**Supplementary Figure 5. Complementing main Figure 4 by showing the same analysis also in trials with away microsaccades and without microsaccades in the window between 150 to 500 ms after cue onset.** Note how, in contrast to toward- and away-microsaccades trials, trials without microsaccades have no reference event to sort the trials by and are here only included for completeness.

Note how these additional analyses corroborated our main findings of (1) a clear N2pc in the traditional 200-300 ms time window in the toward-microsaccade trials, (2) a relative lack of an N2pc in this time window in the away-microsaccade trials, and (3) a severely weekend N2pc in this time window in the no-microsaccade trials.

Though the data from the away- and no-microsaccades trials were overall less clear – likely owing to less trials and to the lack of a reference event to sort the trials by in the no-microsaccade condition – these data were nonetheless re-assuring by showing how the second lateralized negativity (that the reviewer noticed from around 500 ms after cue onset) continues to align to the direction of attention (no matter whether the preceding microsaccade was toward or away) and by showing the expected reversal of the EOG modulation when aligned to the opposite away microsaccades (further reinforcing the suitability of our microsaccade classification).

An additional comment for the authors to consider (although I don't insist) is that when they say that the N2pc is 'modulated' by microsaccades, it sounds contradictory to the claim that the microsaccades do not cause\directly elicit the N2pc. An alternative could be to say that the N2pc amplitude covaries with presence\direction of microsaccades.

Thank you for this reflection. We use “modulate” as a short and convenient way to capture the profound experimental difference (modulation) in the observed N2pc signal when sorting by microsaccade class. Please also note how we immediately disambiguate it from causing the N2pc in our title, and how we extensively clarify what we mean with modulation in our discussion.

Some minor comments:

Whenever you mention eye muscle artifacts, I think you should also mention the possibility of a corneo-retinal dipole movement (which is also apparently ruled out).

Thank you for this excellent and knowledgeable input. We now see that this is another important and related alternative that is also ruled out by the same reasons that rule out eye-muscle artifacts. Accordingly, at every relevant instance where we mentioned eye-muscle artifacts, we now also mention corneo-retinal dipole movement.

In line 308 when you say 'This resonates with recent studies" the reader could be helped by a few words on what these studies found. This is an important context for your study.

Thank you. We agree and now expanded on this by stating: “This resonates with recent studies on complementary neural markers of attention that also reported that microsaccades are not a necessary pre-requisite for neural modulations by attention to occur.”

Line 163 - 'consisted' should be 'consistent'

Thank you for catching this. We now corrected this.

Line 392 - ref 33 is noted twice. "(cf. [33, 33])".

Thank you. We now also corrected this.

Lines 387 - 394 - If I haven't read the comments and your responses to reviewer 2, I would not have understood the argument here. This needs to be clearer

Thank you for also carefully reading our revisions in response to the other reviewers. We now unpack this in a dedicated paragraph that we revised to: “We thus observed an indirect link between the N2pc and the brain’s oculomotor system at the level of the peripheral output of the oculomotor system, as observed in microsaccades. Our findings leave unaddressed the relation between the N2pc and neuronal activity upstream in the brain’s oculomotor system. Relevant oculomotor activity may remain below threshold for microsaccade execution (e.g., activity associated with unfulfilled microsaccades or microsaccade plans) but nevertheless play a critical role in driving the N2pc. Accordingly, in future work, it will be important to supplement our approach with direct recordings in the superior colliculus (cf. [33,50]) to also assess the link between the N2pc and neuronal activity in circuits that govern microsaccade generation [50].”

**Reviewer 2**

The authors have largely answered my questions.

We are pleased to read this, and we remain grateful for your valuable input, as well as of course for your remaining suggestions that we respond to below.

The stuff about N2PC alignment to the stimulus (Fig. 4) is not really surprising/convincing to me. By definition, the n2pc is a stimulus-locked phenomenon, so why would it not remain locked to the stimulus onset in this new analysis? i.e. there are cases where saccades strongly influence visual sensitivity in such a way that the visual response remains clearly time-locked to stimulus onset but still strongly modulated by the saccades. This is, for example, how it happens with saccadic suppression. The stimulus onset occurs peri-saccadically, but the (strongly) modulated visual response to the stimulus remains clearly stimulus-locked. Anyway, I just felt that this needs to be clarified.

Thank you for this valuable reflection that has helped us to better clarify this point in our article.

Because the reported modulation in microsaccade direction also aligns in time to the cue stimulus, our primary question in Figure 4 was whether the N2pc – as a common marker of spatial attention – is *more* aligned to the cue stimulus or *more* to microsaccades that follow the cue. Indeed, it remains possible that prior reports of stimulus-locked N2pc responses were, in fact, directly driven by (directionally biased) microsaccades that also align to cue-stimulus timing. This possibility is indeed a key motivation behind our study and analyses as we now also more explicitly state when introducing the results in Figure 4: “[…] Indeed, though the N2pc is typically considered as an attentional ERP component that is aligned in time to the onset of attentional cues and/or target stimuli, it remains possible that the N2pc is more aligned to the precise timing of the corresponding attentional modulation in microsaccades. […]”.

To address this possibility, we leveraged trial wise variability in the timing between cue-stimulus onset and microsaccade onset. This showed how the N2pc remains more consistently locked in time to cue-stimulus onset. This is important additional information *also* because it rules out that the reported link is a direct consequence of eye-muscle artifacts or retinal displacement.

Having clarified this, we fully agree that it is still interesting that we found such a pronounced modulation in the cue-stimulus-locked attentional N2pc component by microsaccade presence and direction. This remains a key take-away from our study as we make explicit at key moments in our article.

By the way, reference 31 is completely wrong and much less relevant to the current study! The actual correct reference should be: Zhang, T., Tian, X., Malevich, T., Baumann, M. P., & Hafed, Z. M. (2024). Foveal action for the control of extrafoveal vision. BioRxiv. I certainly hope that the authors read it, because it's much more relevant to the current study, and also especially because the authors wrote in their response letter that they actually read it.

Thank you for catching this. We did, in fact, read the correct reference with great interest and even discussed it in a lab meeting. We had, however, by accident put the wrong citation in the article when inserting it with our reference manager. We now corrected this, thank you.

**Reviewer 3**

The authors have addressed all of my concerns, and I find the manuscript ready for publication. Congratulations on an excellent study!

We are pleased to read this and remain grateful for your valuable input.
